# Effects of nitric oxide inhibitors in mice with bladder outlet obstruction

**DOI:** 10.1590/S1677-5538.IBJU.2015.0441

**Published:** 2017

**Authors:** Marcy Lancia Pereira, Carlos Arturo Levi D’ancona, Julio Alejandro Rojas-Moscoso, Antonio Celso Saragossa Ramos, Fabiola Zakia Mónica, Edson Antunes

**Affiliations:** 1 Departamento de Cirurgia, Faculdade de Ciências Médicas - UNICAMP, Campinas, SP, Brasil;; 2 Departamento de Urologia, Faculdade de Ciências Médicas - UNICAMP, Campinas, SP, Brasil;; 3 Departamento de Farmacologia, Faculdade de Ciências Médicas - UNICAMP, Campinas, SP, Brasil

**Keywords:** NG-Nitroarginine Methyl Ester, Nitric Oxide, Urinary Bladder, Ureteral Obstruction

## Abstract

**Purpose:**

To investigate the lower urinary tract changes in mice treated with L-NAME, a non-selective competitive inhibitor of nitric oxide synthase (NOS), or aminoguanidine, a competitive inhibitor of inducible nitric oxide synthase (iNOS), after 5 weeks of partial bladder outlet obstruction (BOO), in order to evaluate the role of constitutive and non-constitutive NOS in the pathogenesis of this experimental condition.

**Materials and Methods:**

C57BL6 male mice were partially obstructed and randomly allocated into 6 groups: Sham, Sham + L-NAME, Sham + aminoguanidine, BOO, BOO + L-NAME and BOO + aminoguanidine. After 5 weeks, bladder weight was obtained and cystometry and tissue bath contractile studies were performed.

**Results:**

BOO animals showed increase of non-voiding contractions (NVC) and bladder capacity, and also less contractile response to Carbachol and Electric Field Stimulation. Inhibition of NOS isoforms improved bladder capacity and compliance in BOO animals. L-NAME caused more NVC, prevented bladder weight gain and leaded to augmented contractile responses at muscarinic and electric stimulation. Aminoguanidine diminished NVC, but did not avoid bladder weight gain in BOO animals and did not improve contractile responses.

**Conclusion:**

It can be hypothesized that chronic inhibition of three NOS isoforms in BOO animals leaded to worsening of bladder function, while selective inhibition of iNOS did not improve responses, what suggests that, in BOO animals, alterations are related to constitutive NOS.

## INTRODUCTION

Nitric oxide (NO) is synthesized from its precursor L-arginine via NO synthases (NOS), which exist in three isoforms: neuronal (nNOS), endothelial (eNOS) and inducible (iNOS). The first ones are constitutively expressed and produce small quantities of NO and the last one is induced by cytokines, infection or other stimuli and produces large amounts of NO. Mice obstructed for 5 weeks exhibit morphologic and functional disorders and these changes were attributed to enhanced expression of iNOS early after obstruction, which would be responsible for improving oxygenation during obstruction-induced ischemia (1). Although NO can be produced by several sources, including endothelial cells, nerves, smooth muscle and urothelium, studies demonstrated that major sites of NO release were urothelium and afferent nerves (2).

Treatment of BOO rats with aminoguanidine, a competitive inhibitor of iNOS, has shown good results, as decreases in iNOS ameliorated functional and fibrotic changes in the bladder (3, 4). The same consequences have been observed in iNOS knockout mice (1, 4). Treatment with L-NAME, a non-selective competitive inhibitor of NOS, inhibited generation of nitrotyrosine, which is produced by nitrogen reactive species and, as consequence, improved bladder contraction (5). However, another study showed that a feeding diet rich in L-arginine was beneficial for rabbits with 2 weeks of severe BOO (6).

In the current study, we investigated lower urinary tract changes in mice treated with L-NAME or aminoguanidine after 5 weeks of BOO, since these drugs represent non-selective and selective NOS inhibitors, respectively.

## MATERIALS AND METHODS

### Animals and Experimental Groups

The experimental protocols were approved by the Ethical Principles in Animal Research adopted by the Brazilian College for Animal Experimentation (COBEA, No 2030-1). Male C57BL6 mice (25-30g), 8-9 weeks old, were used and randomly allocated into six experimental groups: Sham (Sham-operated), Sham + L-NAME (Sham that received L-NAME), Sham + aminoguanidine (Sham that received aminoguanidine), BOO (bladder outlet obstruction), BOO + L-NAME (BOO that received L-NAME) and BOO + aminoguanidine (BOO that received aminoguanidine). Doses of L-NAME (150mg/Kg) and aminoguanidine (20mg/Kg) were chosen according to previous study (7). All animals were placed into individual cages with food ad libitum and received drugs given in the drinking water immediately after surgery for a period of 5 weeks, when all in vitro and in vivo studies were performed.

### Surgical Procedures

Animals were anesthetized by intraperitoneal injection of ketamine (2mg/Kg) and xylazine (30mg/Kg) and placed in the supine position. A lower midline abdominal incision was made and, after exposure of the bladder and proximal urethra, partial BOO was created by tying a 6-0 nylon suture around the urethra. A 0.6mm diameter tubing was used as a guide to prevent total urethral occlusion. In Sham group, identification of bladder and proximal urethra was done, with no further surgical manipulation. Both abdomen muscles and skin were closed with a 6-0 nylon suture.

In vivo and in vitro bladder functional assessment

### Bladder weights

Bladders were carefully withdrawn and weighted. Also, mice were weighted for normalization of values and the ratio bladder weight (mg) to body weight (g) was obtained. This parameter was used to verify if BOO animals exhibited bladder weight increase, showing that pathological alterations due to this condition, imposed by surgery, has occurred.

### Filling Cystometry

Animals were anesthetized by intraperitoneal injection of urethane (1.2g/Kg) and a lower midline incision was made to expose the bladder. A 25-gauge butterfly cannula was inserted into the bladder dome and was connected via a 3-way stopcock to a pressure transducer (AD Instruments, Sydney-NSW, Australia) and infusion pump (Harvard Apparatus, Holliston, MA). After bladder emptying, saline solution at room temperature was infused at a rate of 0.6mL/h until detrusor equilibration and then, counted for 30 minutes after the end of the first micturition cycle. Pressure registers were obtained by a Powerlab 4/30 data acquisition (6.0 system, AD Instruments, Sydney-NSW, Australia). Maximum detrusor pressure was defined as the peak of intravesical pressure during a micturition. Bladder capacity was determined as the total infused volume immediately before the first micturition. Compliance was measured as bladder capacity divided by the maximum detrusor pressure minus baseline pressure. Non-voiding contractions (NVC) were defined as increases in intravesical pressure (>4mmHg) not associated with release of urine through urethra. Micturition frequency was measured considering the number of micturition cycles within 30 minutes. Threshold pressure was defined as the pressure immediately before micturition (8). Bladders of mice used for cystometry were not used in the other experiments.

### Tissue bath

Animals were killed by inhalation of CO_2_. Bladders were rapidly dissected, removed, weighted and put immediately in a physiologic saline solution of Krebs-Henseleit (NaCl 117mM, NaHCO_3_ 25mM, C_6_H_12_0_6_ 11mM, KCl 4.7mM, NaHPO_4_ 1.2mM, MgSO_4_7H_2_O 1.2mM and CaCl_2_2H_2_O 2.5mM, pH 7.4). Two longitudinal muscle strips with intact urothelium were obtained from the bladder body, measuring 2 x 2 x 10mm and were mounted in 10mL organ baths containing Krebs-Henseleit solution at 37ºC bubbled with a gas mixture of 95% O_2_ and 5% CO_2_. Changes in isometric force were recorded using a computer Software (PowerLab v.7.0 system, AD Instruments, Sydney-NSW, Australia). The resting tension was adjusted to 5mN at the beginning of the experiments, the equilibration period was 60 minutes and the bathing medium was changed every 15 minutes.

Cumulative concentration-response curves to the full muscarinic agonist Carbachol (1ƞM to 30μM) were constructed using one-half log unit. Nonlinear regression analysis to determine the pEC_50_ was carried out using Graph Pad Prism (Graph Pad Software, Inc., San Diego, CA, USA) with the constraint that F=0. All concentration–response data were evaluated for a fit to a logistics function in the form: E = E_max_ / ([1 + (10c/10x)n] + F), where E is the maximum response produced by agonists; c is the logarithm of the EC_50_, the concentration of drug that produces a half-maximal response; x is the logarithm of the concentration of the drug; the exponential term, n, is a curve-fitting parameter that defines the slope of the concentration–response line, and F is the response observed in the absence of added drug. Data were normalized to the wet weight of the respective urinary bladder strips, and the values of E_max_ were represented in mN/mg.

Bladder strips were stimulated with Electric Field Stimulation (EFS at 2, 4, 8 and 16Hz), 50V, during 0.2ms and an interval of 2 minutes between pulses. Responses were recorded and maximal tensions compared, expressed by mN/mg.

### Statistical analysis

The values recorded were mean±SEM, and the statistical significances were determined by One-Way ANOVA followed by the Tukey test. P<0.05 was considered significant. InStat© was used for statistical analysis.

### Drugs

No-Nitro-L-arginine methyl ester hydrochloride (L-NAME), urethane, Carbachol and aminoguanidine hemisulfate were obtained from Sigma Chem. Co. (St Louis, MO).

## RESULTS

### Bladder weights

**Figure 1:**
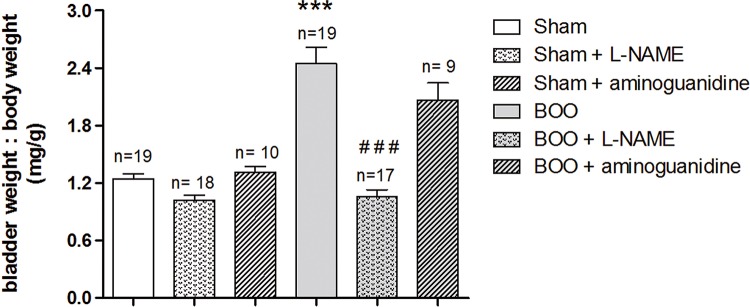
Bladder weight to body weight ratio in Sham and BOO mice treated or not with L-NAME or aminoguanidine for 5 weeks.

Bladder outlet obstruction (BOO) animals showed increased bladder weight to body weight ratio (P<0.001), almost 2-fold when compared to Sham animals. Oral treatment with L-NAME in BOO animals decreased bladder weight to body weight (P<0.001). Treatment with aminoguanidine had no significant effect on this parameter (Figure-1).

***P<0.001 versus Sham. # # # P<0.001 versus BOO.

### Filling Cystometry

**Figure 2:**
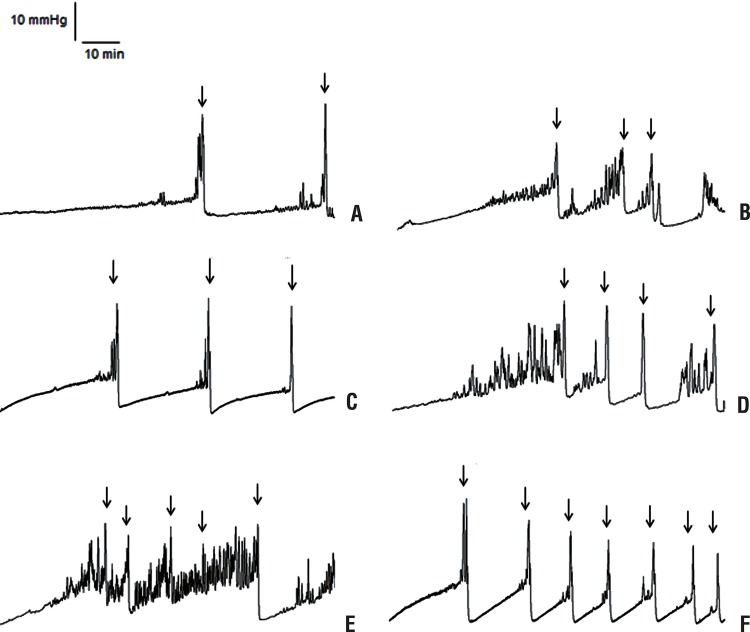
Representative Cystometrograms in Sham (A), Sham + L-NAME (B), Sham+aminoguanidine (C), BOO - Bladder outlet obstruction (D), BOO + L-NAME (E) and BOO + aminoguanidine (F) groups. Arrows indicate micturition peaks.

A total of 9 Sham, 6 Sham + L-NAME, 6 Sham + aminoguanidine, 13 BOO, 6 BOO + L-NAME and 7 BOO + aminoguanidine were evaluated. Figure-2 represents cystometric profiles obtained after 5 weeks of surgical procedures for all experimental groups.

**Figure 3:**
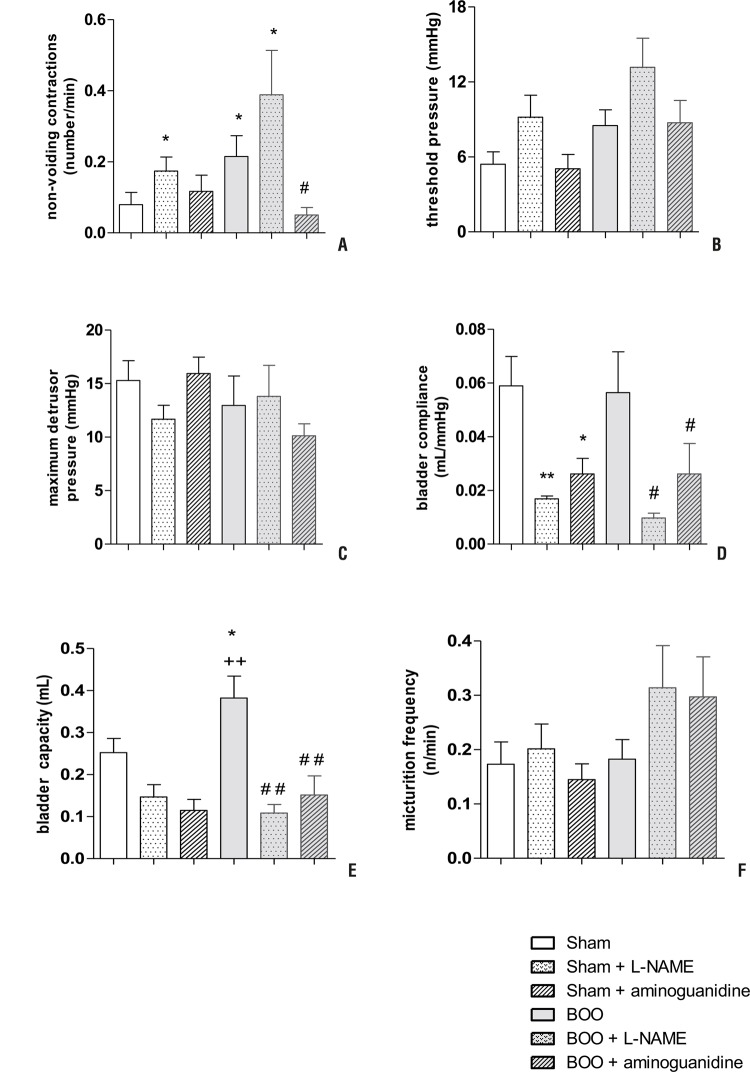
Cystometric parameters in Sham and BOO mice treated or not with L-NAME or aminoguanidine for 5 weeks: non-voiding contractions (A), threshold pressure (B), maximum detrusor pressure (C), compliance (D), bladder capacity (E) and micturition frequency (F).

BOO animals showed more NVC and higher compliance than Sham. Treatment with L-NAME increased non-voiding contractions and diminished bladder capacity and compliance in BOO animals. BOO mice treated with aminoguanidine showed less NVC and lower bladder capacity. Although a tendency of increase in micturition frequency caused by L-NAME and aminoguanidine in BOO mice was observed, there were no statistical differences for this parameter, for maximum detrusor pressure or threshold pressure. Sham mice treated with aminoguanidine showed lower bladder capacity (Figure-3 and Table-1).

* P<0.05 versus Sham; ** P<0.01 versus Sham; # P<0.05 versus BOO;

## P<0.01 versus BOO; ++ P<0.01 versus Sham + L-NAME.

**Table 1:** Cystometric parameters evaluated for each experimental group. NVC: non-voiding contractions.

Experimental Group	NVC (n/min)	Micturition frequency (n/min)	Threshold pressure (mmHg)	Maximum detrusor pressure (mmHg)	Bladder capacity (mL)	Compliance (mL/mmHg)
Sham (n=9)	0.079 ± 0.034	0.173 ± 0.041	5.406 ± 0.985	15.288 ± 1.859	0.252 ± 0.034	0.059 ± 0.011
Sham + L-NAME (n=6)	0.174 ± 0.039	0.201 ± 0.046	9.168 ± 1.753	11.673 ± 1.306	0.147 ± 0.029	0.017 ± 0.001 **
Sham + aminoguanidine (n=6)	0.117 ± 0.045	0.145 ± 0.029	5.045 ± 1.146	15.933 ± 1.542	0.115 ± 0.026	0.026 ± 0.006 *
BOO (n=13)	0.215 ± 0.059 *	0.183 ± 0.036	8.496 ± 1.294	12.965 ± 2.741	0.382 ± 0.052 * ^++^	0.056 ± 0.015
BOO + L-NAME (n=6)	0.388 ± 0.125 *	0.314 ± 0.078	13.155 ± 2.329	13.808 ± 2.905	0.108 ± 0.021 ^# #^	0.001 ± 0.001 ^# #^
BOO + aminoguanidine (n=7)	0.050 ± 0.021 ^#^	0.297 ± 0.074	8.717 ± 1.785	10.133 ± 1.114	0.151 ± 0.046 ^# #^	0.026 ± 0.011 ^# #^

* P<0.05 versus Sham; # P<0.05 versus BOO; # # P<0.01 versus BOO; ++ P<0.01 versus Sham + L-NAME.

### Tissue bath

**Figure 4:**
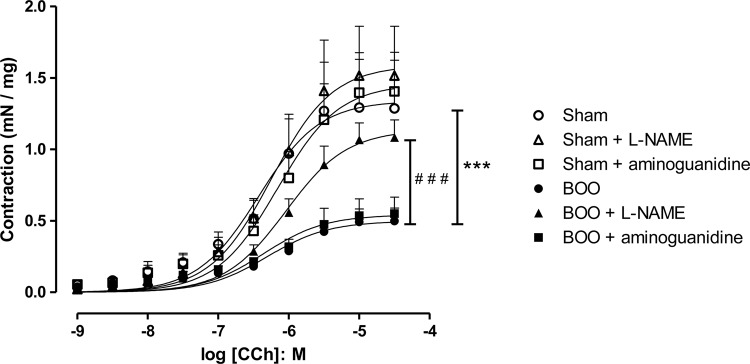
Bladder smooth muscle contractions to Carbachol in Sham and BOO mice treated or not with L-NAME or aminoguanidine for 5 weeks.

Cumulative addition of the muscarinic agonist Carbachol (1ƞM to 30μM) to bladder preparations produced concentration-dependent contractions (Figure-4 and Table-2). Sham + L-NAME mice showed higher responses compared to Sham. BOO and BOO + aminoguanidine groups exhibited a significant decrease in maximal responses to this agonist. The pEC_50_ values for Carbachol did not change significantly for any group.

*** P<0.001 versus Sham. # # # P<0.001 versus BOO.

**Table 2:** Maximal responses (Emax) and Potency (pEC50) to Carbachol (Cch) in bladder smooth muscle. Data were obtained in Sham and BOO animals, treated or not with L-NAME or aminoguanidine.

Experimental group	Emax (mN/mg)	pEC50
Sham (n=9)	1.29 ± 0.13	6.43 ± 0.21
Sham + L-NAME (n=3)	3.06 ± 0.40***	6.38 ± 0.17
Sham + aminoguanidine (n=4)	1.40 ± 0.27	6.15 ± 0.16
BOO (n=10)	0.49 ± 0.09*	6.30 ± 0.17
BOO + L-NAME (n=6)	1.08 ± 0.12 +++#	6.04 ± 0.10
BOO + aminoguanidine (n=3)	0.55 ± 0.11*	6.35 ± 0.17

* P<0.05 versus Sham; *** P<0.001 versus Sham; # P<0.05 versus BOO. +++ P<0.001 versus Sham + L-NAME.

**Figure 5:**
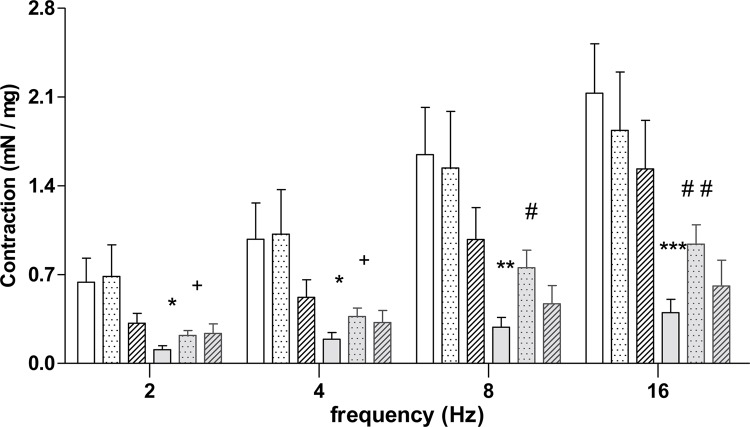
Contractile responses to electrical-field stimulation (EFS; 2-16 Hz) in bladders from Sham and BOO mice, treated or not with L-NAME or aminoguanidine for 5 weeks.

EFS induced a frequency-dependent increase in the amplitude of contractions in isolated bladder smooth muscle. BOO animals showed lower contraction when compared to Sham animals at all frequencies evaluated. L-NAME BOO treated animals showed decreased contraction at 2 and 4Hz compared to Sham + L-NAME and increased contraction compared to BOO mice at 8 and 16Hz. There was no difference for aminoguanidine treated animals (Figure-5 and Table-3).

* P<0.05 versus Sham; ** P<0.01 versus Sham; *** P<0.001 versus Sham; # P<0.05 versus BOO; # # P<0.01 versus BOO; # # # P<0.001 versus BOO; + P< 0.05 versus Sham + L-NAME.

**Table 3:** Contraction (mN) produced by electric stimulation in isolated bladder smooth muscle.

Experimental Group	2 Hz	4 Hz	8 Hz	16 Hz
Sham (n=9)	0.639 ± 0.190	0.977 ± 0.288	1.645 ± 0.372	2.129 ± 0.388
Sham + L-NAME (n=5)	0.686 ± 0.249	1.018 ± 0.352	1.539 ± 0.445	1.836 ± 0.459
Sham + aminoguanidine (n=4)	0.316 ± 0.077	0.520 ± 0.138	0.976 ± 0.252	1.532 ± 0.382
BOO (n=10)	0.108 ± 0.031 *	0.191 ± 0.052 *	0.285 ± 0.077 **	0.398 ± 0.106 ***
BOO + L-NAME (n=8)	0.220 ± 0.038 +	0.369 ± 0.066 +	0.754 ± 0.139 #	0.939 ± 0.153 # #
BOO + aminoguanidine (n=3)	0.236 ± 0.076	0.322 ± 0.095	0.469 ± 0.143	0.610 ± 0.203

* P<0.05 versus Sham; ** P<0.01 versus Sham; *** P<0.001 versus Sham; # P<0.05 versus BOO; # # P<0.01 versus BOO; + P<0.05 versus Sham + L-NAME.

## DISCUSSION

Despite the high prevalence of benign prostatic hyperplasia among men, the mechanisms responsible for voiding dysfunction induced by bladder outlet obstruction (BOO) are not well understood. Research and development of appropriate therapies include the use of animal models in order to understand the physiological control of urinary continence, as well as pathophysiological conditions involved in bladder dysfunction (9).

Several studies used rats (3, 4), mice (1, 10, 11), rabbits (12), guinea pigs (13) and pigs (14) to mimic BOO, whose structural and physiological changes of the bladder wall are similar to those observed in men suffering from BPH (9).

We used an established animal model of BOO. We found that BOO mice showed increased bladder weight to body weight ratio, almost 2-fold when compared to Sham animals (2.45±0.17 and 1.25±0.05, respectively), according to 2.5-fold observed in a study with female mice (10). In fact, mice 1 week after obstruction already have a rapid increase in bladder mass, which worsened 3 and 5 weeks after the surgical procedure (11). Similarly, rats and rabbits have increased blood flow after 24 hours of obstruction, which could be the first stimulus for hypertrophy (12, 15). This event occurs due to stretch of the bladder wall components, leading to thickening of epithelium, muscle layer and serosa (16) and increase in synthesis and deposition of collagen (17). After initial bladder function compensation, blood flow tends to diminish (18) and, as the process becomes chronic, more hypoxic-reperfusion areas can be observed in muscular layer (19). The increased wall thickness and wall tension result in cyclical ischemia-reperfusion during and subsequent to each voiding contraction (19, 20) and cause progressive deterioration of bladder function (17). The oxidative stress increases the production of reactive oxygen species (ROS), leading to enhanced malondialdehyde (MDA) and diminished superoxide dismutase (SOD). Free radicals originating from ischemia-reperfusion injury are one of primary etiologies in obstructed bladder dysfunction (21). A 4-week BOO female mice study revealed increased edema and lymphocytic infiltrate in the lamina propria, and hypoxia was seen in the urothelium, lamina propria and detrusor (5). Six-week BOO male investigation showed increase in muscular hypertrophy and fibrosis (22).

Treatment with the non-selective NOS inhibitor L-NAME showed decrease in bladder weight. In rats treated for 4 weeks with the same drug, morphometric studies showed increased thickness of trigone smooth muscle without affecting detrusor smooth muscle (DSM) thickness (23). Moreover, although early administration of L-NAME enhances ischemic damage at the beginning of the obstructive process, it inhibits the generation of nitrotyrosine and results in preservation of nerve density, reducing initial free radical damage associated with BOO (5). On the other hand, at chronic obstruction, L-NAME also prevents increases in blood flow and ROS generation from the compromised mitochondria, but nitrotyrosine production enhances and leads to worsen in bladder function (24).

In contrast, aminoguanidine treatment did not induce significant reduction of bladder weight in BOO mice, which is in contrast to another study (3), when 2 weeks BOO iNOS knock out and aminoguanidine treated rats presented less fibrosis. In both 3 and 6 week obstructed rats, eNOS activity decreases when compared to Sham animals, but nevertheless is higher than iNOS activity (21). Hypertrophied rat obstructed bladders exhibited weak nNOS expression after 3 and 6 weeks (3). In our study, this indicate that inhibition of iNOS alone did not affected bladder gain mass, unlike inhibition of the three isoforms of NOS had significant results.

Urodynamic characteristics normally correlate with bladder weight, but detrusor overactivity in BOO animals may develop without an increase in bladder weight, suggesting that major disturbances caused by BOO may lie in the afferent signaling pathway (25). Some authors propose that NO may be involved in the regulation of the threshold for bladder afferent firing (26). Moreover, the effects of urothelial NO may not be mediated by a direct action on smooth muscle, because DSM lack soluble guanylate cyclase (GC), an important component in NO-mediated relaxation (27, 28).

Although maximum detrusor pressure and capacity did not reveal significant differences between BOO and Sham animals, the first parameter tended to increase, following compliance, that was higher in BOO mice. This is in agreement with studies with BOO rats (4, 29-31), but is different from what has previously been demonstrated in the obstructed mouse bladder (9, 11). Moreover, BOO mice had more NVC than Sham, similarly to another studies with mice and rats (4, 11, 31). In the unstable bladder, alterations of the smooth muscle can be a consequence of the “patchy denervation” of the detrusor (32), mediated by oxidative stress, since reduced amount of glutathione, the most abundant non-protein thiol with antioxidant capacity, was observed in BOO bladders (33). Predominance of protrusions junctions and ultra-close cell abutments in BOO (34) lead to more sensibility and lose of synchronism, what features detrusor overactivity (3).

L-NAME BOO treated animals had more NVCs than BOO, suggesting worsening of bladder function. The same result was found in chronically L-NAME treated rats (35) and was due to DSM super sensitivity to muscarinic agonists via increases in the levels of [^3^H] inositol phosphate (IP_3_), accompanied by reduction of β_3_-adrenoceptor-mediated DSM relaxations (23). Aminoguanidine treatment decreased NVC in BOO mice, as shown in BOO rats treated with the same drug, due to attenuation in fibrosis. Studies using iNOS knockout mice instead of aminoguanidine treatment present concordance of results, in which NO contributes to ischemic injury and inhibition of iNOS is of therapeutic benefit (4).

Although with no significant difference in micturition frequency for L-NAME and aminoguanidine BOO treated groups, tendency of increase in this parameter was according to the smaller bladder capacity observed for these animals.

In vitro muscle physiology studies showed that BOO strips were able to generate less tension in response to cholinergic stimulation and EFS. Other authors have already demonstrated that bladder function maintains stable until 3 weeks post obstruction, but after 5 weeks there is impairment in detrusor function (1, 11). Unstable human bladders frequently show patchy denervation of the muscle bundles. Some muscle fascicles may be completely denervated, while neighboring bundles appear normal. Other regions may show intermediate innervation. The areas of reduced innervation become infiltrated with connective tissue (36). Guinea-pig obstructed bladders also present denervation, diminished response to EFS and muscarinic agonists (33).

Aminoguanidine treated animals had no role in bladder contraction evaluated by tissue bath experiments. However, L-NAME leaded to higher contraction in BOO mice. Obstructed rabbits treated with L-NAME presented higher bladder contraction to Carbachol and EFS 3 and 7 days post obstruction, in addition to less generation of nitrotyrosine and, consequently, reduction in membrane damage. This could account for the augmentation of contractile function and higher nerve densities observed for these animals (5). Another research with BOO rabbits treated with L-NAME showed reduced apoptosis due to ischemia-reperfusion and had significantly increase in contractile responses compared with non-treated animals (37). In rats chronically treated with L-NAME, it was observed that DSM relaxations mediated by β_3_-adrenoceptors reduced, what suggests that prolonged NO deficiency leads to an overactive bladder (23).

## CONCLUSIONS

L-NAME resulted in less bladder mass gain. The higher contractile responses and the increment in NVC suggests that the overactivity already presented by the BOO animals became worse. Although aminoguanidine treatment had decreased NVC, it leaded to reduced bladder capacity, did not prevent the increase in bladder weight and was not effective in ameliorating contractile responses. Taking the results together, it can be hypothesized that chronic inhibition of three NOS isoforms in BOO animals leaded to worsening of bladder function, while selective inhibition of iNOS did not improve responses, what suggests that, in BOO animals, alterations are related to constitutive NOS.
